# Surface energy balance of the Sygyktinsky Glacier, south Eastern Siberia, during the ablation period and its sensitivity to meteorological fluctuations

**DOI:** 10.1038/s41598-021-00749-x

**Published:** 2021-10-28

**Authors:** Eduard Y. Osipov, Olga P. Osipova

**Affiliations:** 1 Limnological Institute SB RAS, Irkutsk, 664033 Russia; 2 V.B. Sochava Institute of Geography SB RAS, Irkutsk, 664033 Russia

**Keywords:** Climate change, Cryospheric science, Hydrology

## Abstract

The physically based melt of the low elevation Eastern Siberian glaciers is poorly understood due to the lack of direct micrometeorological studies. We used an automatic meteorological station to record the meteorological and energy characteristics of the Sygyktinsky Glacier, south Eastern Siberia (56.8° N, 117.4° E, 2,560 m a.s.l.), during two ablation seasons and computed the surface energy balance (SEB) for 30-min intervals. The glacier ablation was both modeled and measured by stakes and a thermistor cable. The net radiation (R_net_) was the main contributor (71–75 W m^−2^, 89–95%) to the SEB (79 W m^−2^, 100%), followed by sensible (2–4 W m^−2^, 3–5%) and latent (2–3 W m^−2^, 2–4%) heat fluxes. The net shortwave radiation was the main positive component of R_net_, while the net longwave radiation was weak and either negative (− 15 W m^−2^ in 2019) or positive (4 W m^−2^ in 2020). The small proportion of turbulent fluxes in the SEB is explained by the low wind speed (1.2 m s^−1^). The glacier ablation was found to be more sensitive to changes in shortwave radiation and wind speed, suggesting the need to consider the atmospheric conditions of the ablation period (summer snowfalls, cloudiness, wind speed) when analyzing long-term trends in glacial changes.

## Introduction

The summer melt of mountain glaciers is an important component of their mass balance and, together with winter accumulation, controls the spatial change of the glaciers. To understand the physical processes that determine the intensity of glacial ablation, an approach associated with the assessment or modeling of all components of the surface energy balance (SEB) on the glaciers is widely used. Such studies are carried out using automatic weather stations (AWSs) on glaciers located on different continents and in different geographic settings, for example, in Scandinavia^1–4^, the European Alps^5,6^, the Caucasus^7^, the Tibetan Plateau and the Himalayas^8–12^, Mongolian Altai^13^, Africa^14,15^, North America^16^, South America^17,18^, New Zealand^19^, Antarctica^20^, and Greenland^21^. However, little is known about the SEB on the glaciers located in northern Asia and especially in Eastern Siberia as yet. This is explained by the remoteness of these glaciers and the lack of systematic meteorological and glaciological observations. Sparse data on the SEB components, as a rule, refer to the mid 1950–60 s (studies conducted within the framework of the International Geophysical Year) and testify to the important role of solar radiation in glacier melt^22,23^. However, the methodological approaches implemented in these studies are now outdated. Moreover, these studies were mostly short-term, and the observation periods did not overlap with each other. This makes it difficult to conduct a qualitative comparative analysis of the physical mechanisms of summer ice melting.

Since July 2019, we have been conducting continuous field meteorological studies on the Sygyktinsky Glacier, one of the largest glaciers of the Kodar Ridge, in south Eastern Siberia. The studies include high-resolution measurements of meteorological and radiation characteristics both on the glacier itself and on its terminal moraine. The data obtained make it possible to compute a model of the energy fluxes determining the summer melt of the glacier with acceptable accuracy. In this paper, we present the meteorological characteristics and the SEB of the glacier during two ablation seasons, 2019 and 2020.

**Figure 1:**
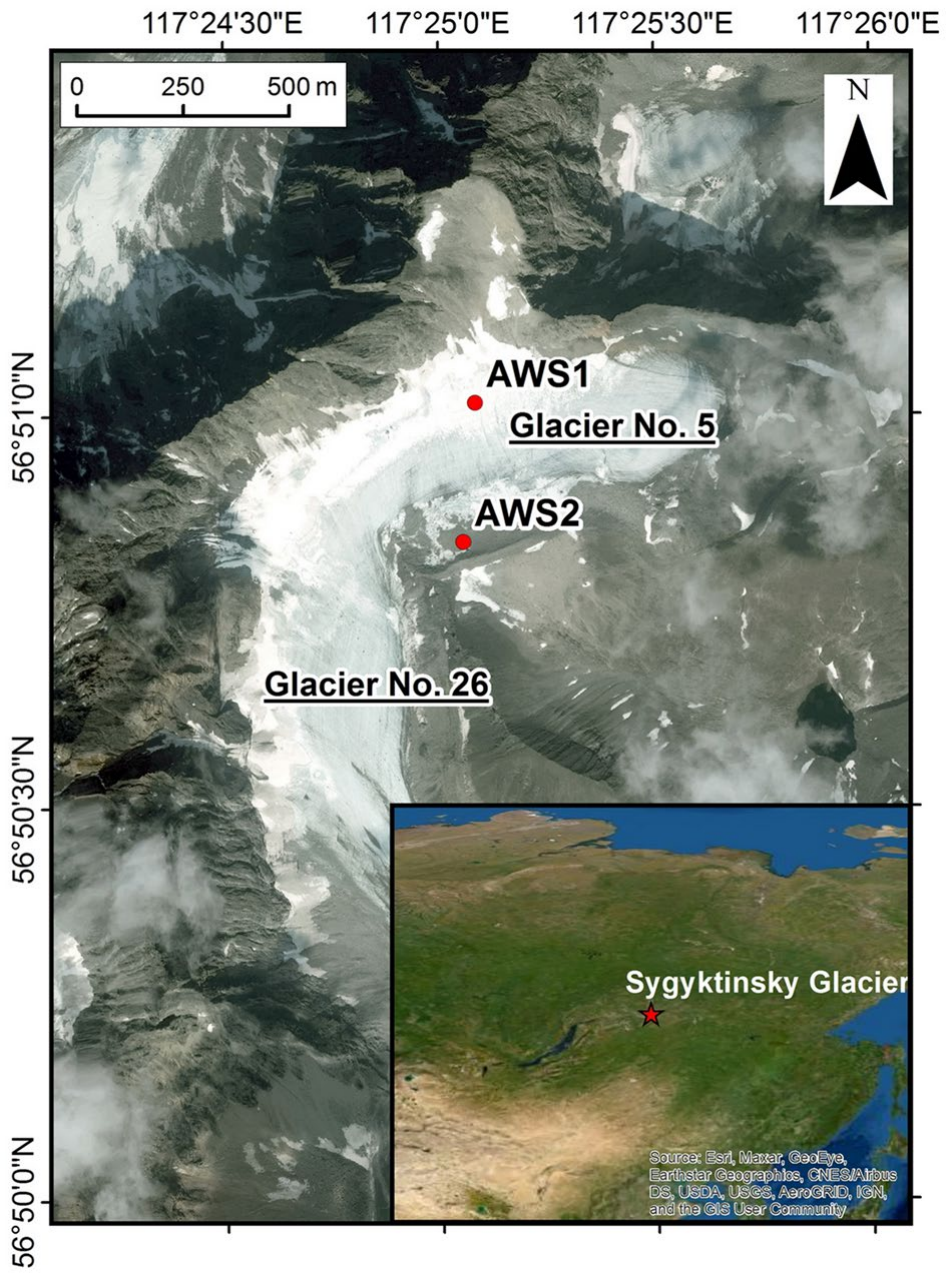
Sygyktinsky Glacier and locations of automatic weather stations AWS1 and AWS2. The satellite image IRS-P5 from 20 August 2009 was provided by SCANEX. The inset map in the lower right corner was obtained from the Google Earth (Google Earth Pro for desktop version 7.3.4, https://www.google.com/earth) as a screenshot.

The Kodar Ridge (the highest summit, “Peak BAM”, is 3072 m a.s.l.) is located in Transbaikalia, in the southern part of Eastern Siberia (Fig. 1). The central part of the ridge is a local glaciation center of Eastern Siberia, which includes 36 small glaciers with a total debris free area^24^ of 9.12 km^2^. The first glaciological survey and catalog of the Kodar glaciers was made in the late 1950s^25^. Since the end of the Little Ice Age, the Kodar glaciers have shrunk the most in Eastern Siberia, on average by 60%^24,26^. Glacier changes in the Kodar region are mainly related to the summer temperature increase (by 1.8–2.2 °C from 1970 to 2010)^24^.

The Sygyktinsky Glacier is the only transection glacier on the Kodar, located in two river basins, Levaya Sygykta and Syulban (Fig. 1). The glacier consists of two branches, the southern (glacier No. 26) and eastern (glacier No. 5) branches^27^. Field micro-meteorological studies were carried out on glacier No. 5 and its terminal moraine. Glacier No. 5 has an eastern aspect, although due to its asymmetry, the glacier surface is more inclined to the southeast (Table S1). In the north and northeast, the glacier is bounded by a watershed ridge up to 2988 m high. The glacier is mainly fed by avalanches from the slopes with southeastern and southern aspects. The glacier tongue is relatively steep (up to 30°) and has no debris cover. The Little Ice Age terminal moraine (up to 50 m high) is well defined and located 220–350 m from the glacier terminus^28,29^.

The climate of the Kodar region can be described based on the Chara meteorological station (711 m a.s.l.), located ~ 50 km east from the glacier (Fig. S1). The temperature and precipitation records of the Chara station are characterized by statistically significant correlations with those from high-mountain central Kodar area^28^. Climate conditions (averaged for the 1961–1990 period) are characterized by a frosty and dry winter (− 28.4 °C, 13 mm) and a warm and humid summer (13.8 °C, 210 mm). The mean annual air temperature is − 7.7 °C, and the mean annual precipitation is 342 mm. Temperatures in 2019 and 2020 were higher than the long-term average in all seasons (except for autumn 2019). The mean summer temperature was 1.7 °C and 0.9 °C above the long-term average in 2019 and 2020, respectively. The summer precipitation in 2019 was 120 mm lower than the long-term average, while in 2020, it was 40 mm higher. In winter, the Asian anticyclone dominates here, but it is a low baric formation, and its influence is indistinct in the Kodar highlands (> 2000 m a.s.l.). Summer (June–August) precipitated moisture is advected either with Arctic invasions or tropical air masses from the southwest and southeast^30^.

## Results

**Figure 2:**
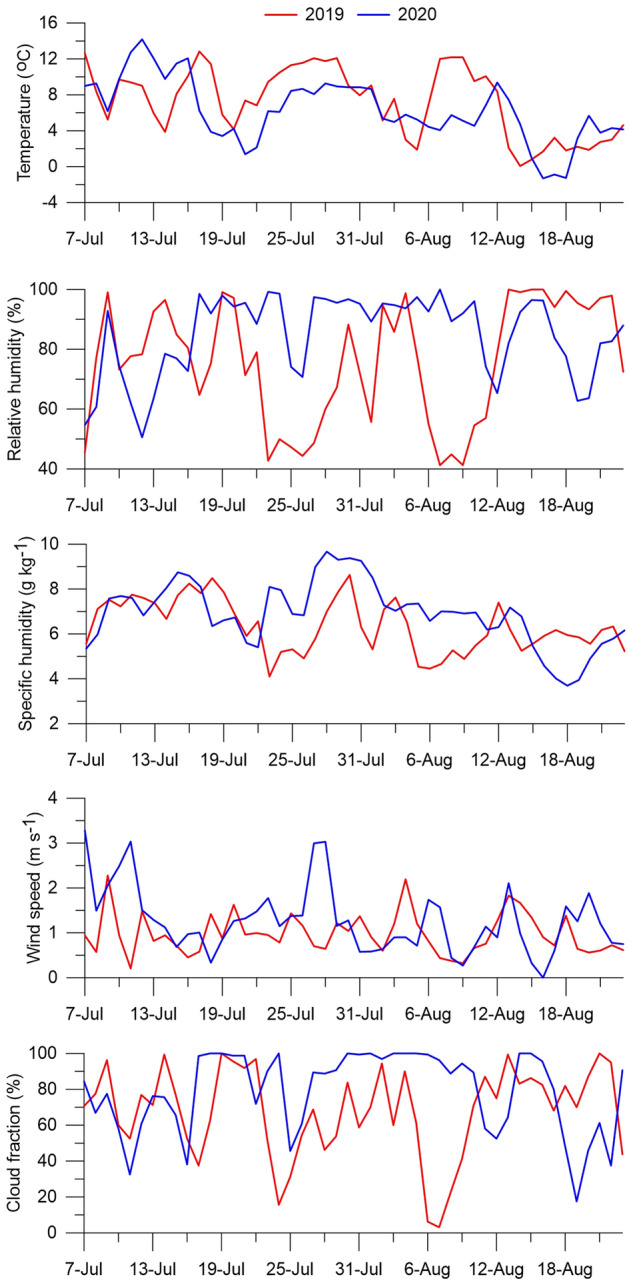
Mean daily values of temperature, relative humidity, and wind speed on the glacier during the 2019 and 2020 ablation seasons (for the period 7 July to 23 August). Cloudiness data were obtained at the Chara weather station.

The meteorological conditions averaged for two ablation seasons (for the same period from July 7 to August 23) are shown in Table 1, and daily mean values of the measured parameters are shown in Fig. 2.

**Table 1:** Mean values of meteorological and radiation parameters on the glacier for the period 7 July to 23 August.

Parameter	2019	2020	Mean	Change
Air temperature, T (°C)	7.3	6.3	6.8	− 1.0
Relative humidity, RH (%)	76.0	85.1	80.6	9.1
Precipitation, P (mm)	119.4	272.0	195.7	152.6
Specific humidity, q (g kg^−1^)	6.4	6.9	6.7	0.5
Atmospheric pressure, p (hPa)	749.1	748.1	748.6	− 1.0
Wind speed, u (m s^−1^)	1.1	1.3	1.2	0.2
Maximal wind speed, u_m_ (m s^−1^)	3.5	4.0	3.8	0.5
Wind direction (°)	–	269.0	–	–
Incoming shortwave radiation, S_in_ (W m^−2^)	157.8	125.3	141.6	− 32.5
Reflected shortwave radiation, S_ref_ (W m^−2^)	67.8	58.3	63.1	− 9.5
Incoming longwave radiation, L_in_ (W m^−2^)	300.6	319.2	309.9	18.6
Outgoing longwave radiation, L_out_ (W m^−2^)	315.6	315.6	315.6	0.0
Sensible heat, H (W m^−2^)	2.1	4.2	3.2	2.1
Latent heat, LE (W m^−2^)	1.3	3.1	2.2	1.8
Atmospheric transmissivity, τ	0.37	0.31	0.34	− 0.06
Albedo (accumulative), α_acc_	0.43	0.47	0.45	0.04
Total cloudiness, C_tot_ (%)	67.5	78.8	73.2	11.3
Low cloudiness, C_low_ (%)	44.4	58.1	51.3	13.7

Cloudiness data were obtained at the Chara weather station.

### Air temperature and humidity

The air temperature (AWS1) during the 2019 ablation season was in 96% of cases above the ice melting point (0 °C) and ranged from − 3.2 to 20.4 °C (on average 7.3 °C, standard deviation 3.8 °C). In 2020, the summer period was cooler: the temperature was positive in 93% of cases and ranged from − 4.5 to 21.0 °C (on average 6.3 °C, standard deviation 3.6 °C). From July to August, the air temperature decreased in both seasons. The surface temperature was approximately 0 °C (melting snow and ice). The temperature gradient between the glacier surface and the 2 m level was positive, on average 3.1 °C m^−1^. This indicates the predominance of stable conditions in the boundary atmospheric layer (temperature inversion).

**Figure 3:**
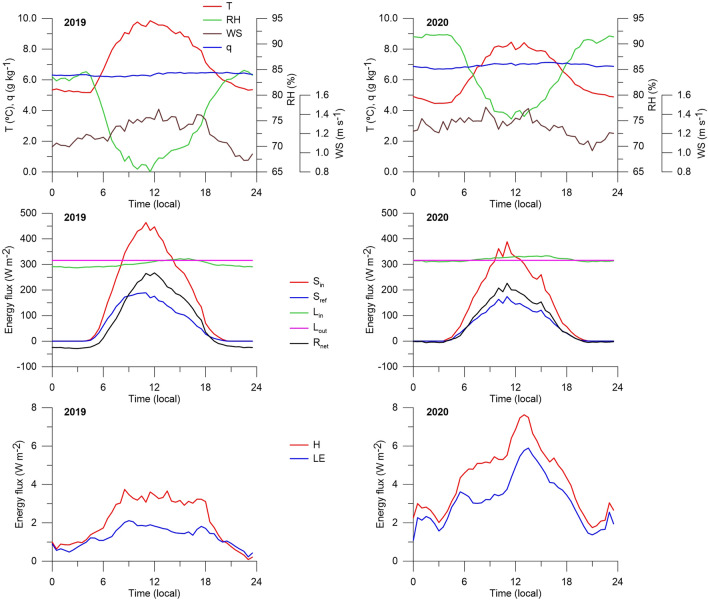
Mean daily cycle of meteorological parameters (2 m above the glacial surface) and energy fluxes during the 2019 and 2020 ablation seasons (for the period 7 July to 23 August): air temperature (T), relative humidity (RH), wind speed (WS), specific humidity (q), incoming (S_in_) and reflected (S_ref_) shortwave radiation, incoming (L_in_) and outgoing (L_out_) longwave radiation, net radiation (R_net_), and sensible (H) and latent (LE) heat.

The diurnal cycle of temperature fluctuates in the range of about 4 °C and has a pronounced maximum in the afternoon (Fig. 3). The minimum temperature is observed during the night (between 3:00 and 4:30). The daily cycle of relative humidity varies in the range of 17% and has a minimum in the afternoon. The specific humidity has a small daily cycle (within 0.4 g kg^−1^) with a maximum in the afternoon (between 15:00 and 18:00).

The average relative air humidity at 2 m above the glacier surface (AWS1) was 76 ± 20% in 2019 and 85 ± 14% in 2020 (Table 1). In 2019, the daily humidity varied from 41 to 100%, and in 2020, from 51 to 100%. Fluctuations in humidity with a frequency of several days are due to a change in synoptic processes over the region (maxima correspond to cyclones, and minima to anticyclones). The daily specific humidity in 2019 ranged from 4.1 to 8.6 g kg^−1^, and in 2020, from 3.7 to 9.7 g kg^−1^ (Fig. 2). In both seasons, there is a tendency for the specific humidity to decrease from July to August.

### Wind speed and direction

The wind regime on the glacier (AWS1) is characterized by the predominance of weak winds (Table 1). In 2020, daily average wind speeds at 2 m above the glacier surface ranged from 0 to 3.3 m s^−1^, with an average of 1.3 m s^−1^ (standard deviation 0.8 m s^−1^). The observed half-hour wind speed reached 8.0 m s^−1^ (Fig. S2), and the maximum wind speed reached 19.1 m s^−1^. The average and maximum wind speed on the glacier (AWS1) were, respectively, 20% and 10% higher than on the moraine (AWS2), due to the increased openness of AWS1. The correlation between the wind speed on the glacier and moraine is statistically significant but not high (R^2^ = 0.35, n = 2247, p < 0.001). The correlation between the maximum wind speeds is higher (R^2^ = 0.52, n = 2247, p < 0.001).

The prevailing wind direction on the glacier (AWS1) is defined by synoptic processes and local topography (Fig. S2). Westerly wind directions (W + WSW + WNW, 248–293°) have the highest occurrence (30%) and higher speeds, and they are associated with large-scale atmospheric circulation. Over the glacier, at the level of 700 hPa, southwestern winds prevailed (25%) during the observation period of 2020. There is a pronounced secondary maximum of the northerly wind (occurrence 10%), which is weak and flowing down from the surrounding mountain slopes. The low-frequency periodicity in the maximum wind speed (5–8 days) is associated with the passage of atmospheric fronts (Fig. 2).

The wind has a weakly expressed daily cycle with increased values in the first half of the day and decreased values in the second (Fig. 3). The maximum speed values are observed in the morning (8:30) and in the afternoon (13:00), and the minimum in the evening (21:00).

### Cloudiness

The summer period at the Kodar is characterized by overcast conditions and rainy weather due to increased cyclonic activity^30^. In 2020 the cloudiness (data from the Chara weather station) was higher than in 2019 and had less variability (Table 1, Fig. 2). In 2019, the cloudiness ranged from 3 to 100% (mean value is 68%, standard deviation 25%), and in 2020 from 18 to 100% (79% on average, standard deviation 23%). The highest cloud cover was associated with cyclones, and the lowest with anticyclones. The daily averaged cloud cover in Chara is statistically significantly correlated (2019: R^2^ = 0.53, n = 48, p < 0.001; 2020: R^2^ = 0.62, n = 48, p < 0.001) with the cloudiness over the glacier, calculated as the ratio between shortwave radiation incoming at AWS1 and at the top of the atmosphere S_TOA_^18^.

### Energy fluxes

#### Shortwave radiation

**Figure 4:**
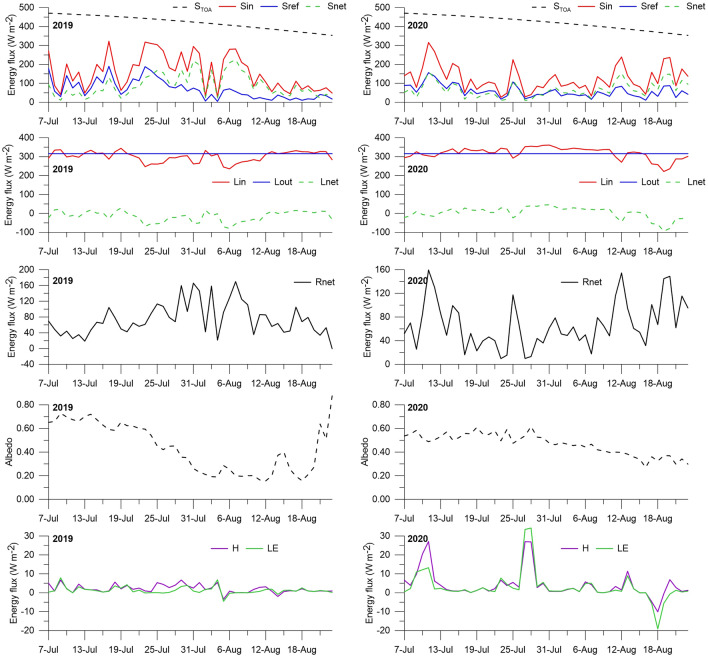
Mean daily values of radiation and turbulent energy fluxes and albedo during the 2019 and 2020 ablation seasons (for the period 7 July to 23 August): top of atmosphere radiation (S_TOA_), incoming (S_in_) and reflected (S_ref_) shortwave radiation, incoming (L_in_) and outgoing (L_out_) longwave radiation, net radiation (R_net_), sensible (H) and latent (LE) heat fluxes.

The incoming summer shortwave radiation (S_in_) at the glacier (AWS1) was low due to increased cloudiness related to cyclonic activity over the study area (Table 1). The atmospheric transmissivity is 0.34 on average, i.e., only one third of the solar radiation entering the top of the atmosphere reached the glacial surface. S_in_ has a strong variability, both day-to-day and interannual (Fig. 4). In 2019, its daily values fluctuated between 28 and 323 W m^−2^ (on average 158 W m^−2^, standard deviation 91 W m^−2^), and in 2020, between 26 and 316 W m^−2^ (on average 125 W m^−2^, standard deviation 67 W m^−2^). The average S_in_ in July 2019 was 65 W m^−2^ higher than in August 2019, while the values in July and August 2020 were approximately equal. In general, the ablation season is characterized by a decreasing trend in S_in_ in accordance with the general decrease in solar radiation arriving at the top of the atmosphere. The lower amount of incoming solar radiation in 2020 compared to 2019 (21% less) is explained by more significant (17% more) cloud cover in 2020 (Table 1). S_in_ has a distinct diurnal cycle (Fig. 3), with the daily range in 2019 being 1.2 times higher than in 2020.

Reflected shortwave radiation (S_ref_, AWS1) is characterized by strong day-to-day variability and tends to decrease towards the end of the ablation season due to an increase in the glacial surface albedo (Fig. 4). At the same time, its interannual variability is relatively small (Table 1), since AWS1 is located near the long-term equilibrium line of the glacier, and the change of the snow surface from snow to ice occurs every year.

The net shortwave radiation ranged from 11 to 225 W m^−2^ in 2019 (on average 90 W m^−2^) and from 11 to 160 W m^−2^ in 2020 (on average 67 W m^−2^). The net shortwave radiation in August was higher than in July (in 2019 by 7 W m^−2^, in 2020 by 20 W m^−2^) due to a decrease in the reflected radiation in August (in 2019, by 3.4 times; in 2020, by 1.5 times).

#### Albedo

From July to August 2019, albedo decreased linearly from 0.73 to 0.19 (Fig. 4). The decrease is associated with snow melting, dust deposition on the glacier surface from adjacent slopes, and the melting out of fine-grained material. The snow cover completely melted on 4 August. The background albedo values in August were low (about 0.20); however, pronounced peaks in August (0.40 to 0.88) marked the summer snowfalls (e.g., 14–15 and 21–23 August). The duration of the periods with high albedo was no more than 2–3 days, indicating that the snow melted quickly. In general, the 2019 ablation season is characterized by two different albedo regimes, reflecting different physical conditions of the glacier surface: (i) a gradual linear-like decrease in albedo in July (snow) and (ii) low background values with pronounced peaks of short-term summer snowfalls in August (ice). The average albedo in the 2019 ablation season was 0.40 (0.57 in July, 0.29 in August). A stable (winter) snow cover on the glacier surface began to form on 6 September.

At the beginning of July 2020, due to the higher winter accumulation, the thickness of the snow cover on the glacier near AWS1 was 2.5 times (4.7 times in w.e.) more than at the beginning of July 2019. Furthermore, the snow cover completely melted here only at the end of August. Accordingly, the change in albedo in 2020 differed from that in 2019 (Fig. 4). If in July, the albedo fluctuated within the narrow range of 0.47–0.62 (on average 0.54), then in August, it had a pronounced decreasing trend from 0.48 to 0.30 (on average 0.39). During the study period, summer snowfalls were observed on 28 July and 19–20 August (small peaks on the curve). In general, the average albedo in the AWS1 area in July was 1.4–2 times higher than in August.

### Longwave radiation

The sensors of longwave radiation were installed only on the moraine (AWS2) to prevent the risk of losing them due to snow avalanches failing down from surrounding slopes to glacier surface. Since during both ablation seasons the glacier surface was melting (about 0 °C) the emitted longwave radiation flux was taken constant (316 W m^−2^)^11^. Both weather stations (AWS1 and AWS2) are located close to each other (300 m in distance and 30 m in altitude) within the same boundary layer, therefore we assumed the incoming longwave radiation measured off the ice (L_in_, AWS2) was corresponded to that on the glacier (AWS1). Compared to shortwave radiation (Fig. 4), L_in_ had insignificant interannual and day-to-day variability and ranged from 236 to 344 W m^−2^ in 2019 (on average 301 W m^−2^, standard deviation 28 W m^−2^) and from 222 to 361 W m^−2^ in 2020 (on average 319 W m^−2^, standard deviation 31 W m^−2^). It changed slightly within the ablation season and was maximum in July 2020 (328 W m^−2^) and minimum in August 2019 (300 W m^−2^).

However, the net longwave radiation in 2019 was slightly different from that in 2020. In 2019, it varied from − 80 to 29 W m^−2^ (average − 15 W m^−2^), while in 2020, it varied from − 94 to 46 W m^−2^ (on average 4 W m^−2^). That is, if in the 2019 observation period, the net longwave radiation was a sink of energy, then in 2020, on the contrary, the glacier surface received additional energy from the atmosphere. In 2019, the daily net longwave radiation was positive for 20 days (42% of the period), and in 2020, for 31 days (65%) due to increased cloudiness in 2020. The relationship between L_in_ and cloudiness is confirmed by a good correlation between them (R^2^ is 0.52 in 2019 and 0.56 in 2020, n = 48).

L_in_ is characterized by a weak diurnal variation with an afternoon maximum (Fig. 3). Some differences are observed between the diurnal cycles in 2019 and 2020. In 2019, the net longwave radiation was negative in the evening and at night, and close to zero in the daytime. In 2020, it was close to zero in the evening and at night, and positive in the daytime. This indicates a positive contribution of longwave radiation to the glacier melting in 2020 and a negative contribution in 2019. Accordingly, at night and in the evening, the net radiation (R_net_) of the glacier surface in 2019 was negative (the glacier was losing energy), while in 2020, it was close to zero.

### Turbulent fluxes

In contrast to shortwave and longwave radiation, the turbulent fluxes of sensible (H) and latent (LE) heat at the glacier (AWS1) were small (Fig. 4). The total turbulent flux (H + LE) was 27 and 9 times less than S_net_ in 2019 and 2020, respectively. The largest day-to-day fluctuations of turbulent fluxes occurred in the 2020 ablation season. The mean values of H and LE in July were higher than in August. Daily values of H were positive on 90% of days and varied from − 3 to 7 W m^−2^ in 2019 (on average 2 W m^−2^), and from − 10 to 27 W m^−2^ in 2020 (on average 4 W m^−2^). Daily values of LE were positive on 85% of days (condensation predominated) and varied from − 5 to 8 W m^−2^ in 2019 (on average 1 W m^−2^), and from − 19 to 34 W m^−2^ in 2020 (on average 3 W m^−2^). Negative daily LE values (evaporation conditions) were observed only for 5 days in 2019 (23 and 26 July, 5–6 August, and 14 August) and 2020 (15 and 17–20 August). Thus, during both ablation seasons, the glacier received additional heat by sensible (H > 0) and latent (LE > 0, condensation) fluxes. The low H and LE fluxes are explained by the constant prevalence of stable (inversion) conditions (Ri > 0) in the boundary air-glacier layer and extremely low wind speed. The relationship of H and LE with wind speed explains their good correlation with each other (R^2^ from 0.47 in 2019 to 0.82 in 2020, n = 48). High peaks of sensible heat (> 20 W m^−2^) on 10–11 July and 27–28 July in 2020 were associated with a high average daily wind speed (up to 3 m s^−1^, Fig. 2) due to the passage of warm atmospheric fronts over the Kodar.

In the diurnal cycle, the turbulent fluxes of both sensible and latent heat were always positive, with an afternoon maximum (Fig. 3). More pronounced maxima of sensible and latent heat were observed in 2020. In contrast to 2019, the night and evening turbulent fluxes in 2020 fully compensated the energy loss by longwave radiation. In general, during the ablation season, stable (inversion) air conditions prevailed both during the day and at night.

### Surface energy balance and melting

**Figure 5:**
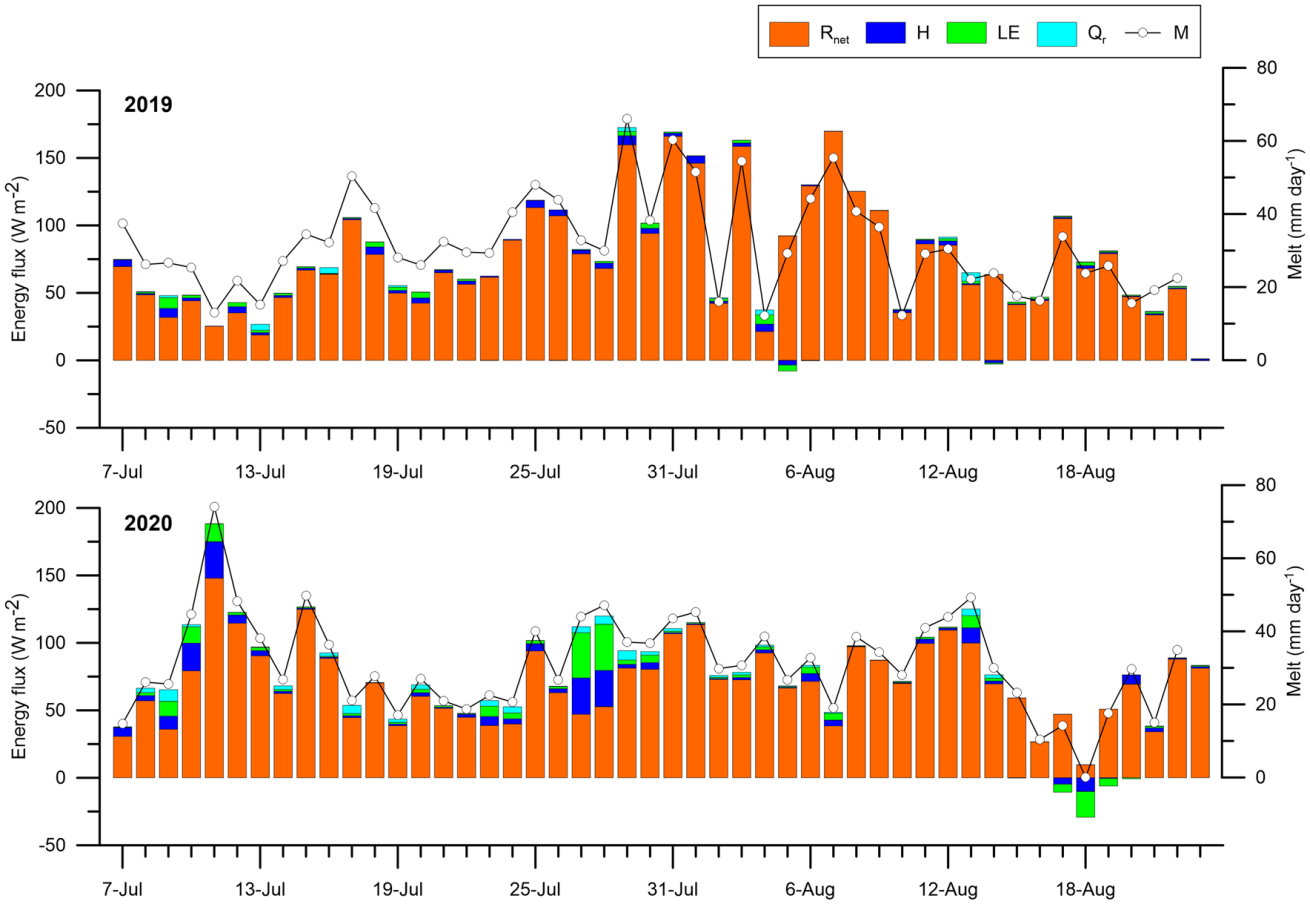
Mean daily values of the SEB components (R_net_, H, LE, and Q_r_ in W m^−2^) and the calculated melt (M in mm day^−1^) for the period from 7 July to 23 August 2019–2020.

The glacial surface was melting for almost the entire observation period, with the exception of night frosts, when all available heat was spent on heating the glacier surface to the melting point (4% of cases in 2019 and 7% in 2020). That is, almost all the heat supplied to the surface was spent on melting snow and ice. The main source of melting energy was R_net_ (89–95%), followed by turbulent heat (H + LE, 5–9%) (Table 2, Fig. 5). The energy input with rainfall Q_r_ varied insignificantly (daily values from 0 to 6 W m^−2^ in 2019, and from 0 to 9 W m^−2^ in 2020) and was an insignificant positive component of the SEB (1–2%). The subsurface heat flux Q_g_ contributed negatively to the SEB and was insignificant (< 1%).

**Table 2:** Components of the surface energy balance (SEB) of the glacier for the period from 7 July to 23 August 2019–2020.

Year	W m^−2^						%					
	SEB	R_net_	H	LE	Q_r_	Q_g_	SEB	R_net_	H	LE	Q_r_	Q_g_
2019	78.6	74.9	2.1	1.3	0.6	− 0.3	100	95.4	2.7	1.6	0.7	− 0.4
2020	79.3	70.7	4.2	3.1	1.6	− 0.3	100	89.1	5.3	4.0	2.0	− 0.4

R_net_ was dominated by the net shortwave radiation S_net_, but its contribution to the SEB differed in 2019 and 2020. If in 2019 S_net_ was 115% and L_net_ − 20%, then in 2020, S_net_ was 85% and L_net_ 4%. That is, if in 2019 the glacier lost the energy for melting due to longwave radiation (− 15 W m^−2^), then in 2020, it received it (4 W m^−2^). S_in_ greatly influenced the day-to-day changes in S_net_, which is confirmed by their high correlation (in 2019, R^2^ = 0.72, n = 48).

The daily values of the SEB were positive on all days, with the exception of 18 August 2020 (Fig. 5). They varied from 0 to 172 W m^−2^ in 2019 (on average 78 W m^−2^), and from − 20 to 188 W m^−2^ in 2020 (on average 79 W m^−2^). In 2019, the SEB in August was slightly higher than in July, while in 2020, on the contrary, it was lower. Despite the higher S_net_ values in 2019 compared to 2020 (90 and 67 W m^−2^, respectively), the mean SEB values in 2019 and 2020 were the same (79 W m^−2^). This is explained by the positive net longwave radiation (4 W m^−2^), increased turbulent heat fluxes (H + LE, 7 W m^−2^) on the background of increased cloudiness, and higher wind speeds in 2020 (Table 1).

### Ablation

In 2019 (48-day period, 7 July to 23 August), the total measured mass balance (ablation) was − 1.38 m w.e. (29 mm w.e. day^−1^), with July and August accounting for − 0.85 and − 0.53 m w.e. (61 and 39%), respectively. The mean daily ablation in July and August differed by 1.4 times (34 and 24 mm w.e. day^−1^, respectively). By the time the snow cover completely melted (4 August 2019), the total ablation was − 1.05 m w.e. (76% of the total ablation). For the same period in 2020, the total ablation was − 1.47 m w.e. (31 mm w.e. day^−1^). The total and daily average ablation in July were slightly higher than in August (− 0.80 and − 0.63 m w.e. and 32 and 29 mm w.e. day^−1^, respectively).

**Figure 6:**
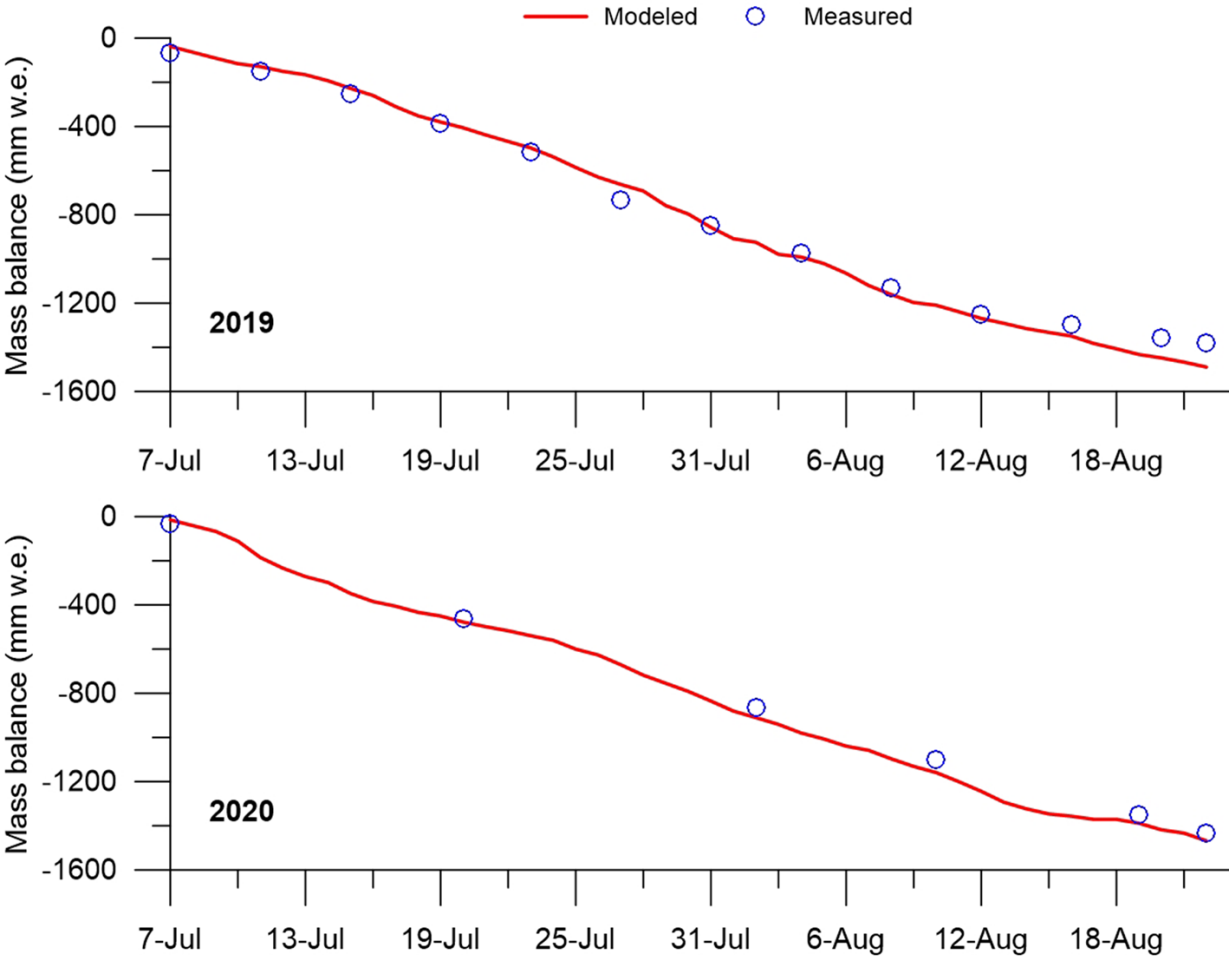
Modeled and measured glacier mass balance (mm in w.e.) for the period from 7 July to 23 August 2019–2020. To minimize the measurement errors, the measured ablation in 2019 was taken as 5-day sums.

The modeled and measured 48-day mass balances were in good agreement both in 2019 and 2020 (Fig. 6). In general, the ablation period was characterized by a more or less uniform decrease in the glacier mass balance. The root-mean square error (RMSE) of the model is 0.01 m w.e., which is within the accuracy of the ablation and SEB measurements. The model slightly overestimates the total ablation. In 2019, the modeled mass balance (− 1.49 m w.e.) was at 0.11 m w.e. more than the measured one (in July and August by 0.01 and 0.10 m w.e., respectively). The largest discrepancy between the modeled and the measured ablation in August can be explained by the influence of summer snowfalls, which could slow down the surface melt. In 2020, the modeled balance (− 1.47 m w.e.) was only 0.04 m w.e. more than measured.

## Discussion

**Figure 7:**
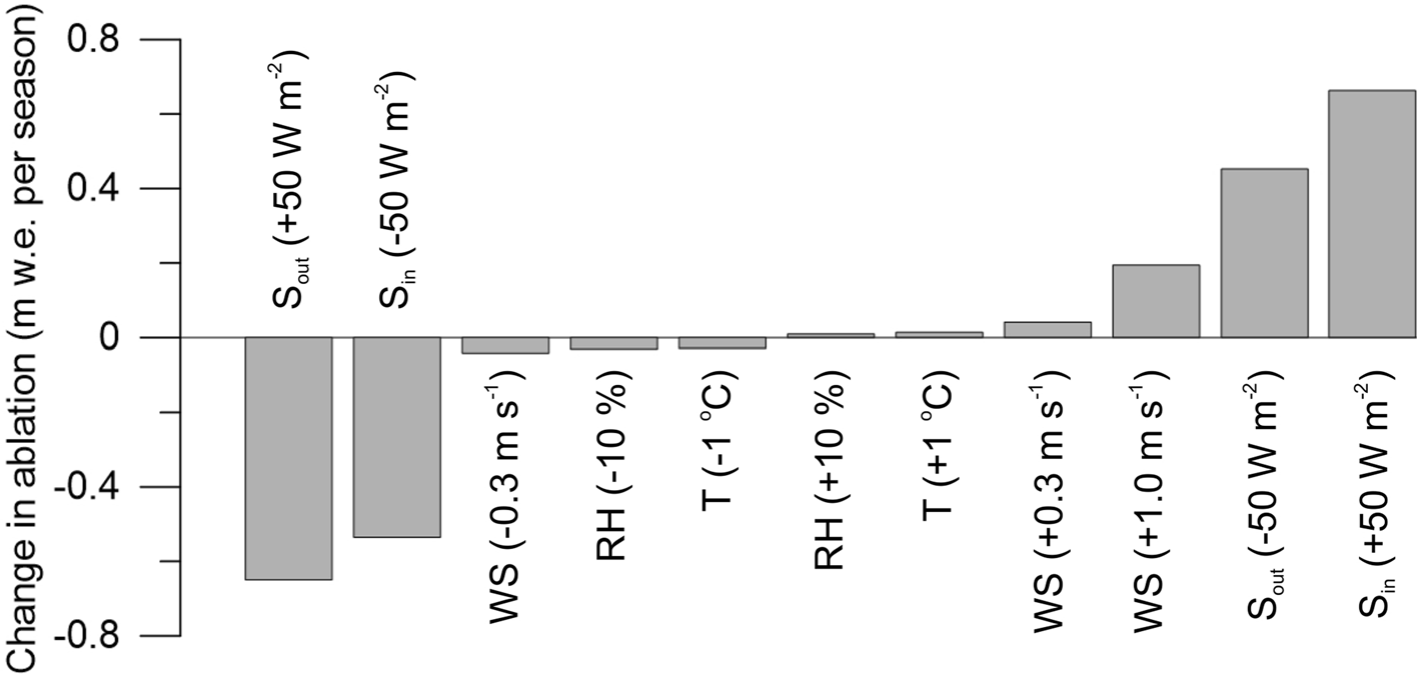
Calculated changes in total ablation for the period 7 July to 23 August (in m w.e.) after perturbations to input parameters: air temperature (T, ± 1 °C), relative humidity (RH, ± 10%), wind speed (WS, ± 0.3 and + 1.0 m s^−1^), and incoming (S_in_, ± 50 W m^−2^) and reflected (S_ref_, ± 50 W m^−2^) shortwave radiation.

To test the sensitivity of the modeled SEB and mass balance to changes in meteorological parameters, we repeatedly ran the model with the changed input meteorological parameters: S_in_ and S_ref_ (± 50 W m^−2^), air temperature (± 1 °C), relative humidity (± 10%), and wind speed (± 0.3 and + 1.0 m s^−1^). The tests showed (Fig. 7) that with an increase in air temperature by 1 °C, the total ablation will increase by only 0.014 m (1.0%), and with a decrease in temperature by 1 °C, ablation will decrease by 0.029 m (1.9%). With an increase in relative humidity of 10%, ablation will increase by 0.01 m (0.7%), and with a decrease, it will decrease by 0.031 m (2.1%). With an increase in wind speed by 0.3 m s^−1^, ablation will increase by 0.042 m (2.8%), and with a decrease by 0.3 m s^−1^, it will decrease by 0.041 m (2.7%). At the same time, with an increase in wind speed by 1 m s^−1^, ablation will noticeably increase by 0.195 m (13%). In our calculations, changes in the air temperature, humidity, and wind speed only affected the turbulent component of the SEB. Sensible heat is sensitive to changes in the wind speed and air temperature, while latent heat is sensitive to changes in the temperature, humidity, and wind speed. An increase in wind speed has the strongest effect on the magnitude of turbulent fluxes and their proportion in the melt energy. For example, with an increase in wind speed by 1 m s^−1^, the turbulent heat fluxes (H + LE) will increase by 3 times, and their proportion will increase to 21%. However, changes in the shortwave radiation have the greatest influence on glacier ablation. With an increase in S_in_ by 50 W m^−2^, the total ablation would increase by 0.664 m (44%), and with a reduction, it would decrease by 0.536 m (36%). With an increase in reflected radiation by 50 W m^−2^, melting would decrease by 0.65 m (43%), and with a decrease, accordingly, it would increase by 0.453 m (30%). S_ref_ has the greatest effect on changes in albedo (from − 0.17 to 0.25, respectively). In general, the summer mass balance of the glacier is most sensitive to changes in the radiation components of SEB (S_in_ and S_ref_, albedo).

We compared the SEB and summer meteorological conditions of the Sygyktinsky Glacier with those of some other mid-latitude (40–65°N) glaciers of Eurasia (Table 3). As the data were obtained in different ablation seasons and using different approaches, the analysis is only very preliminary. However, we can see some similarities and differences in the SEB components of the glaciers and their relations with meteorological conditions. In general, S_in_ decreases with increasing latitude. The relatively low average value of S_in_ at the Kodar (< 200 W m^−2^) is close to that on the glaciers of Southern and Eastern Siberia (Altai, Suntar-Khayata), as well as on the Storbreen glacier in Norway. This is probably due to significant cloudiness, which has maximum values in Kodar, Altai, and Norway (> 70%). Accordingly, the net shortwave radiation on these glaciers (with the exception of Altai) has rather low values (< 100 W m^−2^). All glaciers lost radiative heat by outgoing longwave radiation (L_out_), while due to high cloudiness, the smallest losses were observed on the Storbreen and Sygyktinsky glaciers (< 10 W m^−2^). The contribution of R_net_ to the SEB at Kodar (92%) was slightly higher than on the glaciers in the Alps, Scandinavia, Altai, and Suntar-Khayata (76–88%). Accordingly, the contribution of turbulent heat to the melt energy at the Sygyktinsky Glacier was minimal (8%), while the glacier received additional heat both in sensible and latent (condensation) form. Such an insignificant proportion of turbulent heat on the Sygyktinsky Glacier, despite the high air temperature, is explained by extremely low wind speeds (< 1.5 m s^−1^), the lowest among all compared glaciers (Table 3). In turn, low wind speeds on the glacier are probably explained by the predominance of the low gradient baric field over the Eastern Siberia in summer^30^. It is known that under stable air stratification (dT > 0) at low wind speeds, turbulent heat transfer is suppressed as a result of the action of hydrostatic stability^31^. Studies in Greenland have shown that aerodynamic stability reduces the sensible heat flux over the melting ice surface compared to that predicted for a stable boundary layer^21^. Thus, changes in such meteorological characteristics as cloudiness and wind speed most strongly affect the structure of the SEB and the summer mass balance of the Sygyktinsky Glacier.

**Table 3:** Comparison of the components of surface energy balance and meteorological parameters on several mid-latitude glaciers of Eurasia.

Region (glacier)	Location	Period (years)	S_net_	L_net_	R	H	LE	SEB	T/RH/u/cloud	Reference
			Wm^−2^ (%)	Wm^−2^ (%)	Wm^−2^ (%)	Wm^−2^ (%)	Wm^−2^ (%)	Wm^−2^	°C/%/m s^−1^/%	
French Alps (glacier de Saint-Sorlin)	45° N, 6° E, 2760 m a.s.l	8.07–28.08 (2006)	157 (102)	− 33 (− 21)	124 (80)	28 (18)	2 (1)	154	5.4/70/3.0/48	Six et al.^5^
Norway (Storbreen)	62° N, 8° E, 1570 m a.s.l	1.06–10.09 (2001–06)	92 (81)	− 6 (− 5)	86 (76)	20 (18)	9 (8)	113	5.3/78/3.2/77*	Andreassen et al.^2^
Caucasus Mountains, Russia (Djankuat glacier)	43° N, 43° E, 3000 m a.s.l	1.07–31.08 (2007–15)	168 (51)	− 17 (− 5)	151 (45)	104 (31)	32 (10)	332	7.5/70/3.9/36*	Toropov et al.^7^
Russian Altai (Malyi Aktru glacier)	50° N, 87° E, 3250 m a.s.l	1.07–31.08 (1970)	123 (97)	− 12 (− 9)	111 (88)	20 (16)	− 5 (− 4)	126	8.2/70/2.0/74	Galakhov^23^
Mongolian Altai (Potanin glacier)	49° N, 88° E, 3040 m a.s.l	13.06–14.08 (2007–08)	206 (143)	− 52 (− 36)	154 (107)	8 (6)	− 18 (− 13)	144	3.4/67/3.3/50*	Konya et al.^13^
Western Qilian mountains, China (Laohugou glacier No. 12)	39° N, 97° E, 4550 m a.s.l	1.06–30.09 (2011)	126 (169)	− 45 (− 60)	81 (108)	7 (9)	− 13 (− 17)	75	− 0.4/65/2.0/60*	Sun et al.^10^
**Kodar (Sygyktinsky Glacier)**	**57° N, 117° E, 2560 m a.s.l**	**7.07**–**23.08 (2019–20)**	**79 (99)**	**− 6 (− 7)**	**73 (92)**	**3 (4)**	**2 (3)**	**79**	**6.8/81/1.2/73**	**This study**
Suntar-Khayata (glacier no. 31)	63° N, 141° E, 2230 m a.s.l	1.07–31.08 (1959)	95 (93)	− 15 (− 15)	81 (79)	16 (16)	6 (6)	102	4.0/83/3.8/84	Gavrilova^22^, Koreisha^32^
Kamchatka Peninsula, Russia (Koryto glacier)	55° N, 162° E, 810 m a.s.l	7.08–12.09 (2000)	–	–	43 (32)	59 (44)	31 (23)	133	7.6/–/2.4/60*	Konya et al.^33^

An asterisk (*) indicates the monthly data on total cloudiness from the ERA-Interim reanalysis of the European Centre for Medium-Range Weather Forecasts (https://www.ecmwf.int/). A dash (–) indicates no data.

## Conclusions

Using meteorological data, we computed all energy fluxes on the Sygyktinsky Glacier, Kodar ridge, Eastern Siberia, during two ablation seasons (July–August 2019 and 2020). The data obtained made it possible to calculate the physically based energy-balance model of the glacier. The model includes both the direct measurement of radiation fluxes and calculated turbulent fluxes (aerodynamic method with stability correction). The model is in good agreement with the measured summer mass balance. Sensitivity tests have shown that the SEB is most sensitive to changes in shortwave radiation and weakly sensitive to changes in temperature and humidity. Meanwhile, the turbulent heat fluxes are largely controlled by wind speed. With an increase in wind speed, the proportion of turbulent heat in the SEB increases sharply. Net shortwave radiation is a dominant source (89–95%) of melt energy, and it is strongly controlled by cloudiness and short-term summer snowfalls (in August). The net longwave radiation is weakly negative/positive, depending on the prevailing weather conditions (cloudiness). Sensible and latent turbulent fluxes are positive components of the SEB. However, their contribution is insignificant (≤ 10% balance) due to the low wind speed (on average 1.2 m s^−1^). Nevertheless, on some windy days, turbulent fluxes can reach quite large values. The heat of precipitation and subsurface fluxes are insignificant over the ablation period and compensate for each other. The SEB on the Sygyktinsky Glacier is similar to that on the other mid-latitude glaciers in South and Eastern Siberia, as well as on the Storbreen glacier in Norway. We explain it by the dominant overcast weather conditions during the ablation period. The prevailing influence of R_net_ in the SEB indicates the need to take into account changes in atmospheric processes (cloudiness, summer precipitation, wind speed) when analyzing long-term glacial trends.

## Methods

### Automatic weather stations

Two automatic weather stations (AWSs) were installed on 6 July 2019 on the glacier and its terminal moraine (Fig. 1). AWS1 was installed on a relatively flat surface (≤ 10) near the ice divide of the glacier at 2561 m a.s.l. (56°51.02′ N, 117°25.09′ E), near the long-term average equilibrium line altitude (Table S1). Temperature, humidity, and shortwave radiation (incoming and reflected) sensors were mounted on a vertical mast. As the glacier surface descended, the mast was thrice redrilled; accordingly, the height of the sensors above the glacier surface was corrected and the orientation of the solar radiation sensors was checked. The glacier is quite narrow (about 300 m, Fig. 1) and avalanches falling down to its surface in winter and spring are common. Therefore, to prevent a risk of losing the expensive sensors of incoming and outgoing longwave radiation we permanently installed them on the moraine station (AWS2), at a vertical mast at height 2.5 m above the ground surface (Table S2). A thermistor cable with temperature sensors (a distance of 10 cm away from each other) was installed in the 2.2 m borehole drilled 3 m from AWS1 to measure the snow/ice temperature and daily ablation. In the winter of 2019/20, AWS1 failed due to an avalanche and thermistor cable was destroyed and the mast with sensors was buried under the snow. On 6 July 2020, a tripod was installed at the same place with the same sensors for air temperature and humidity (at heights of 0.5 and 2.0 m above the glacier surface), wind speed and direction (1.0 and 2.0 m), and S_in_ and S_ref_ (2 m). Measurements of air temperature, humidity, and wind speed at two levels were carried out in order to more accurately estimate the turbulent heat fluxes and the roughness length parameter. The tripod was descending with the glacier surface, while the height of the sensors above the glacier surface always remained constant.

AWS2 was installed on the terminal moraine, as a rigid tripod, at an elevation of 2529 m a.s.l. (56°50.84′ N, 117°25.06′ E), in order to calibrate AWS1 records, and to be a backup in the event of an unexpected AWS1 failure (e.g., due to avalanches). AWS2 included sensors for air temperature and relative humidity, atmospheric pressure, wind speed and direction, shortwave and longwave radiation (incoming and outgoing), and ground temperature (at the surface and at a depth of 10 cm). Here, we assumed that the incoming longwave radiation on the moraine (AWS2) was similar to that on the glacier (AWS2). This assumption is based on the following premise. AWS1 and AWS2 are located close to each other in space (300 m in distance and 30 m in altitude). This means that they are located within the same boundary layer of the atmosphere, which is often estimated at values of the order of 50–100 m. Accordingly, this assumption is confirmed by similar meteorological parameters, temperature and humidity, measured at both stations (R^2^ for temperature and relative humidity are 0.89 and 0.91, respectively). On average, in the summer of 2019, the difference in temperature and humidity between AWS1 and AWS2 was only 0.3 °C and 1%, respectively, which is within the measurement error.

The temperature and humidity sensors were naturally ventilated and protected from solar radiation. The sensor readings were recorded in the memory with a 30 min frequency. The instantaneous wind speed was measured every 30 min, while the maximum wind speed was recorded within each 30-min interval. Irkutsk time (GMT + 8) was used as the closest to local time. The AWS data were loaded onto removable memory cards during field observations on 24 August 2019 and 2020. Due to a save error, wind direction data for 2019 was lost. Thus, continuous time series were obtained for the period from 7 July to 23 August during two seasons of ablation (this period is analyzed in the article). At the beginning and end of the observation period, the density of snow and ice was measured in shallow pits near AWS1. Ablation was also measured in the vicinity of AWS1 using 11 plastic rods. The characteristics of the sensors used in both AWSs are listed in Table S2.

### Data treatment

The raw AWS data were thoroughly checked for errors using basic quality control procedures recommended by the World Meteorological Organization (WMO)^34^. In total, ≤ 1% of the original data (solar radiation, temperature, humidity, and wind speed) were rejected. Erroneous sporadic data were removed, and gaps were filled using linear interpolation. The main errors were typical for solar radiation data and caused by the effect of atmospheric precipitation on the upper sensors (especially in mixed or solid form). Since the rain gauge measured only liquid precipitation correctly, 13% of the precipitation data was rejected without filling.

Albedo was calculated as the “accumulative albedo” ${\alpha }_{acc}$ with a 30-min resolution as the ratio of the sums of reflected and incoming shortwave radiation over a 24-h time window^20^:

1 $${\alpha }_{acc}=\frac{\sum {S}_{ref}}{\sum {S}_{in}}$$

The use of “accumulative albedo” instead of the conventional one makes it possible to neutralize the measurement errors of the incoming shortwave radiation^20^. The specific air humidity q (g kg^−1^) was calculated using formulas adopted by the WMO^35^ with the measured values of the atmospheric pressure p (hPa), air temperature T (°C), and relative humidity RH (%) and the calculated value of the water vapor pressure in humid air e (hPa) as inputs:

2 $$\text{q}=\frac{623\text{e}}{\text{p}-0.377\text{e}}$$

3 $$\text{e}=6.112\text{exp}\left(\frac{17.62\text{T}}{243.12+\text{T}}\right)\left(1.0016+0.0000315\text{p}-\frac{0.074}{\text{p}}\right)\frac{\text{RH}}{100}$$

### Surface energy balance model

The surface energy balance (SEB) was computed for 30-min intervals as

4 $$\text{SEB}={\text{S}}_{\text{in}}+{\text{S}}_{\text{ref}}+{\text{L}}_{\text{in}}+{\text{L}}_{\text{out}}+\text{H}+\text{LE}+{\text{Q}}_{\text{r}}+{\text{Q}}_{\text{g}}$$

where S_in_ and S_ref_ are the incoming and reflected shortwave radiation, L_in_ and L_out_ are the incoming and outgoing longwave radiation, H and LE are the sensible and latent heat, Q_r_ is the heat supplied with rain, and Q_g_ is the subsurface heat flux. S_in_ and S_ref_ were directly measured at the glacier (AWS1) while L_in_ was measured at the moraine (AWS2). Since L_out_ was not measured at AWS1 we assumed a constant value (316 W m^−2^) for a melting glacial surface (0 °C)^11^. All terms are taken to be positive when directed towards the surface and expressed in W m^−2^.

The surface melt M (mm day^−1^) was calculated as

5 $$\text{M}=\frac{\text{SEB}}{{\text{L}}_{\text{f}}}$$

where ${L}_{f}$ is the latent heat of fusion (3.30 × 10^5^ J kg^−1^ for snow and 3.35 × 10^5^ J kg^−1^ for ice).

### Turbulent fluxes

A bulk aerodynamic approach based on the Monin–Obukhov similarity theory, including stability correction, was used to calculate turbulent fluxes. This approach is used when calculating turbulent flows from micrometeorological measurements on glaciers in different climatic settings^8,14,17^. This method shows a good correlation of calculated turbulent flows with those measured with eddy-covariance systems^7^. The sensible and latent turbulent fluxes, H and LE, were calculated as

6 $$\text{H}={\text{c}}_{\text{p}}{\uprho }_{0}\frac{\text{p}}{{\text{p}}_{\text{o}}}\frac{{\text{k}}^{2}\text{u}\left(\text{T}- {\text{T}}_{\text{s}}\right)}{\text{ln}\left(\frac{\text{z}}{{\text{z}}_{{0}_{\text{m}}}}\right)\text{ ln}\left(\frac{\text{z}}{{\text{z}}_{{0}_{\text{t}}}}\right)} {\left({\Phi }_{\text{m}}{\Phi }_{\text{t}}\right)}^{-1}$$

7 $$\text{LE}=0.623 {\text{L}}_{\text{v}}{\uprho }_{0}\frac{1}{{\text{p}}_{\text{o}}}\frac{{\text{k}}^{2}\text{u}\left(\text{e}- {\text{e}}_{\text{s}}\right)}{\text{ln}\left(\frac{\text{z}}{{\text{z}}_{{0}_{\text{m}}}}\right)\text{ ln}\left(\frac{\text{z}}{{\text{z}}_{{0}_{\text{h}}}}\right)} {\left({\Phi }_{\text{m}}{\Phi }_{\text{h}}\right)}^{-1}$$

where ${c}_{p}$ is the specific heat capacity for air at constant pressure (1010 J kg^−1^ K^−1^); ${\rho }_{0}$ is the air density at the standard sea level (kg m^−3^); $p$ and ${p}_{0}$ are the atmospheric pressure at glacier and standard sea levels (hPa); $k$ is the von Karman constant (0.38); $u$, $T,$ and $e$ are the wind speed (m s^−1^), air temperature (K), and water vapor pressure in humid air (hPa) at a measurement level z above the glacial surface (2 m); ${T}_{s}$ and ${e}_{s}$ are the glacier surface temperature (273.15 K for a melting surface) and water vapor pressure (6.11 hPa) at the glacial surface level ${z}_{0}$ at 0 °C; ${L}_{v}$ is the latent heat of evaporation of snow/ice for the melting surface (2514 × 10^3^ J kg^−1^); and ${z}_{{0}_{m}}$, ${z}_{{0}_{t}},$ and ${z}_{{0}_{h}}$ are the surface roughness lengths (m) for moment, temperature, and humidity, respectively. Dimensionless stability functions for the moment (${\Phi }_{m}$), temperature (${\Phi }_{t}$), and humidity (${\Phi }_{h}$) were calculated using the bulk Richardson number ${Ri}_{b}$^17^.

8 $$\text{For }{\text{Ri}}_{\text{b }}> 0 (\text{stable conditions}): {{\left({\Phi }_{\text{m}}{\Phi }_{\text{t}}\right)}^{-1}=\left({\Phi }_{\text{m}}{\Phi }_{\text{h}}\right)}^{-1}={\left(1-5{{\text{R}}_{\text{i}}}_{\text{b}}\right)}^{2}$$

9 $${{\text{For }{\text{Ri}}_{\text{b}} < 0 (\text{unstable conditions}): \left({\Phi }_{\text{m}}{\Phi }_{\text{t}}\right)}^{-1}=\left({\Phi }_{\text{m}}{\Phi }_{\text{h}}\right)}^{-1}={\left(1-16{{\text{R}}_{\text{i}}}_{\text{b}}\right)}^{0.75}$$

${Ri}_{b}$ is a dimensionless characteristic of the relationship between the thermal and dynamic factors of turbulence and was calculated between the measurement level $z$ (2 m) and the glacier surface ${z}_{0}$ as^17^

10 $${\text{Ri}}_{\text{b}}=\frac{\text{g}\left(\text{T}-{\text{T}}_{\text{s}}\right)\left(\text{z}-{\text{z}}_{{0}_{\text{m}}}\right)}{{\text{Tu}}^{2}}$$

where $g$ is the acceleration due to gravity (9.8 m s^−2^).

Taking into account that at very low wind speeds ${Ri}_{b}$ takes unrealistically high values (> 1), we used only wind speeds > 0.5 m s^−1^. In addition, we adopted a critical value of ${Ri}_{b}$ equal to 0.4, assuming that when this value is exceeded, (${Ri}_{b}$> 0.4), turbulence ceases completely (H = LE = 0) and the flow becomes laminar^36^. Thus, the influence of the thermal stability of the near-glacial air layer on the turbulence coefficient was taken into account. In unstable air (a decrease in temperature with height), ${Ri}_{b}$ < 0 and the turbulence coefficient is greater than that in stable air, while in stable (inversion) air, ${Ri}_{b}$> 0 and the turbulence coefficient is less than that in unstable air. Stable (inversion) conditions dominated on the glacier during the entire observation period. For example, in summer 2020, stable air conditions (${Ri}_{b}$> 0) were observed in 91% of cases, while unstable conditions (${Ri}_{b}$< 0) were observed in only 9% of cases.

The roughness lengths for the momentum z_0m_ were calculated from the wind speed measurements at upper (2 m) and lower (1 m) levels at AWS1 during the 2020 ablation season. For this, we used data under the near-neutral air conditions (Ri ≈ 0). When using the criterion |Ri|< 0.1 (17% of observations), the median value of z_0m_ was 0.61 mm (6.1 × 10^−4^ m). The roughness lengths for the temperature z_0t_ (4.3 × 10^−4^ m) and humidity z_0h_ (4.9 × 10^−4^ m) were calculated using the calculated z_0m_ and the roughness Reynolds number ${Re}_{*}$ (here, we used ${Re}_{*}$= 2.5) in accordance with the frequently used parameterization^37^

11 $${\text{z}}_{0\text{t}}=\text{exp}\left\{\text{ln}\left({\text{z}}_{0\text{m}}\right)+0.317-0.565\text{ln}\left({\text{Re}}_{*}\right)-0.183{\left[\text{ln}\left({\text{Re}}_{*}\right)\right]}^{2}\right\}$$

12 $${\text{z}}_{0\text{h}}=\text{exp}\left\{\text{ln}\left({\text{z}}_{0\text{m}}\right)+0.396-0.512\text{ln}\left({\text{Re}}_{*}\right)-0.180{\left[\text{ln}\left({\text{Re}}_{*}\right)\right]}^{2}\right\}$$

### Precipitation and subsurface heat fluxes

The heat flux with liquid precipitation ${Q}_{r}$ was calculated as^1^

13 $${\text{Q}}_{{\text{r}}} = \rho _{{\text{w}}} c_{w} r\left( {{\text{T}} - {\text{T}}_{{\text{s}}} } \right)$$

where ${\rho }_{w}$ is the density of water (980 kg m^−3^), ${c}_{w}$ is the heat capacity of water (4190 J kg^−1^ K^−1^), and $r$ is the precipitation rate (m s^−1^). The type of precipitation was determined based on the air temperature T at the measurement level (2 m). When T ≥ 2.0 °C, the precipitation was considered to be liquid (rain) and used in the calculation of ${Q}_{r}$.

The subsurface heat flux ${\text{Q}}_{\text{g}}$ was calculated as^38^

14 $${\text{Q}}_{\text{g}}=-{\text{k}}_{\uptau }\frac{\left({\text{T}}_{\text{g}}-{\text{T}}_{\text{s}}\right)}{\left({\text{z}}_{\text{g}}-{\text{z}}_{0}\right)}$$

where ${k}_{\tau }$ is the thermal conductivity (0.4 W m^−1^ K^−1^ for old snow and 2.2 W m^−1^ K^−1^ for ice), and ${T}_{g}$ is the temperature (K) of the upper glacier layer at a depth ${z}_{g}$ (m) from the surface. For the calculation, we used the data of the temperature-depth profile obtained only in the 2019 ablation season (in 2019/20 winter, the thermistor cable broke down due to the avalanche and did not work). According to these data, the temperature of the glacier at a depth of 2 m was − 1.7 °C.

### Ablation measurements

Continuous ablation measurements were made using the thermistor cable installed in a 2.2 m borehole next to AWS1 with a daily resolution for the period from 6 July to 22 August 2019. The technique of automatic measurement of ablation using a thermistor cable (vertical arrays of temperature sensors) was recently successfully tested on an Italian glacier^39^. The technique is based on the ability to differentiate between sensors buried in ice/snow and sensors exposed to the atmosphere. Sensors located beneath snow cover and within ice show much lower temperature variability compared to exposed sensors due to solar radiation. Thus, the analysis of temperature data makes it possible to identify the location of a sensor (below, above or on the ice/snow surface). Here we used the daily temperature variance calculations for all sensors to estimate their positions relatively glacier surface. The distance between the temperature sensors (10 cm) made it possible to measure the lowering of the glacier surface with a standard error of ± 5 cm^39^. In addition, ablation was measured in the vicinity of AWS1 by 11 stakes (3 readings per season). In 2020, due to the failure of the thermistor cable, aperiodic ablation measurements were made only by stakes (6 readings per season).

## Supplementary Information

Supplementary Information.
