# Supplementary material for: Surface energy balance of the Sygyktinsky Glacier, south Eastern Siberia, during the ablation period and its sensitivity to meteorological fluctuations
Source: Sci Rep. 2021 Oct 28;11:21260. doi: 10.1038/s41598-021-00749-x (PMC8553739; doi:10.1038/s41598-021-00749-x)
Supplement: Supplementary file 1 — Supplementary Information. [file 41598_2021_749_MOESM1_ESM.pdf]

Supplementary information for:

Surface energy balance of the Sygyktinsky Glacier, south Eastern Siberia, during the ablation period and its sensitivity to meteorological fluctuations

Eduard Y. Osipov[1*], Olga P. Osipova[2]

[1] Limnological Institute SB RAS, Irkutsk, 664033, Russia

[2] V.B. Sochava Institute of Geography SB RAS, Irkutsk, 664033, Russia

[*] Corresponding author (eduard@lin.irk.ru)

Figures:

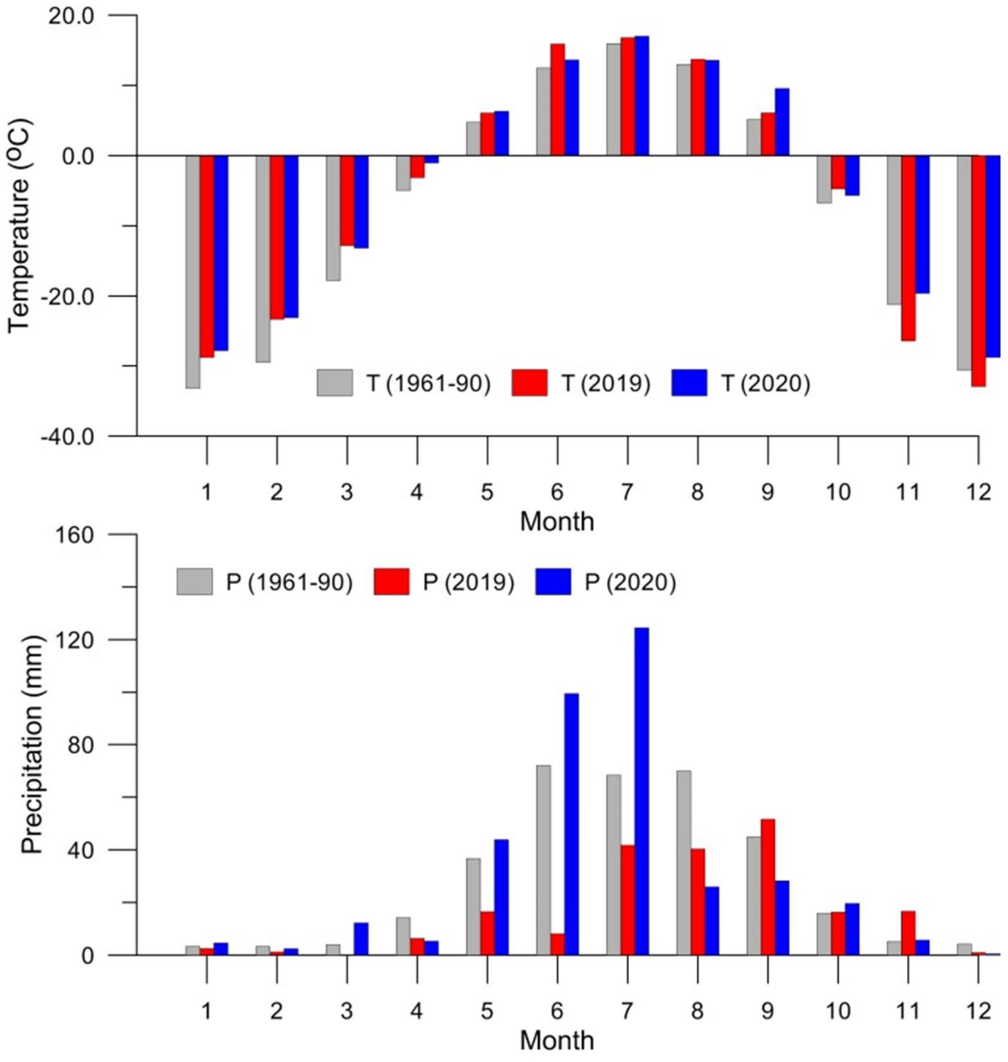

Figure S1: Monthly mean temperature (T) and precipitation (P) data of the Kodar region from the data of the weather station Chara.

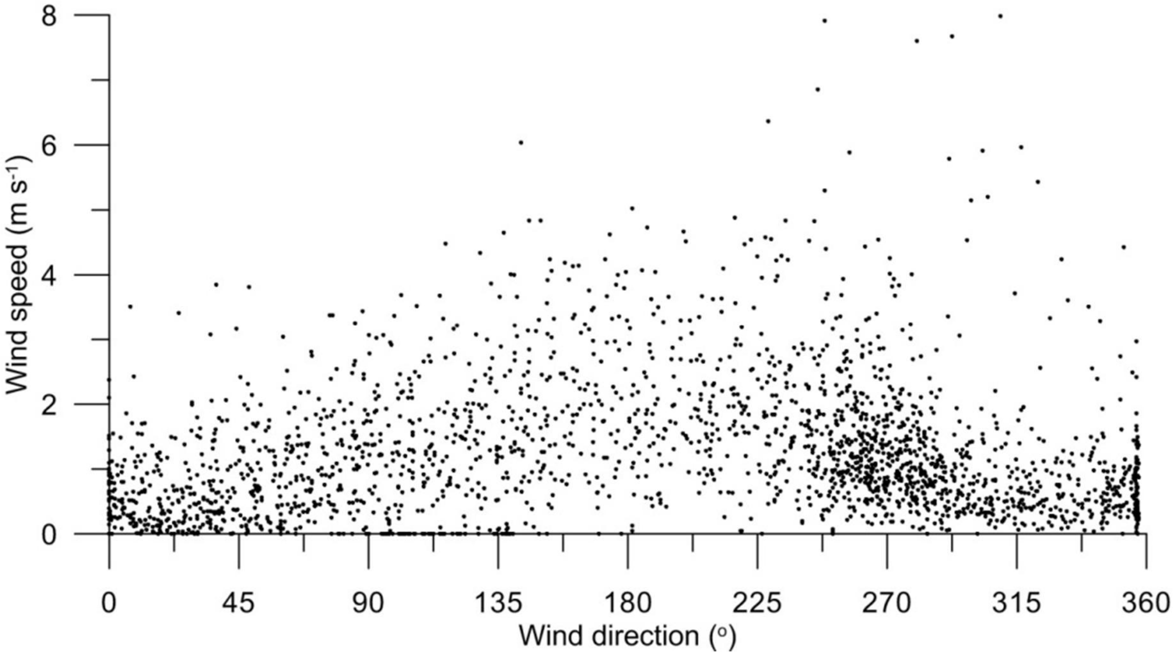

Figure S2: Relationship between wind speed and wind direction at AWS1 (height 2 m). 30-minute data for the period from 7 July to 23 August 2020.

Tables:

Parameter	Value
debris free area	0.291 km^2
length	0.758 km
highest elevation	2,670 m
mean elevation	2,543 m
lowest elevation	2,450 m
mean slope	19°
mean long-term equilibrium line altitude (ELA)	2,535 m

Table S1: Morphometric parameters of eastern branch of the Sygyktinsky Glacier (glacier No. 5)

Parameter	Station	Sensor	Range	Accuracy
air temperature (°C)	AWS1, AWS2	Davis 6830	–40 to +65 °C	±0.3 °C
relative humidity (%)	AWS1, AWS2	Davis 6830	0 to 100 %	±2 %
wind speed (m s^{-1})	AWS1, AWS2	Davis 6410	1 to 89 m s^{-1}	±5 %
wind direction (°)	AWS1, AWS2	Davis 6410	1 to 360 °	±3 °
atmospheric pressure (hPa)	AWS2	Davis	540 to 1,100 hPa	±1 hPa
shortwave radiation, 400–1100 nm (W m^{-2})	AWS1, AWS2	Davis 6450	0 to 1,800 W m^{-2}	±5 %
shortwave radiation, 300–2800 nm (W m^{-2})	AWS2	LPNET14	0 to 2,000 W m^{-2}	±2.6 %
longwave radiation, 4.5–45 µm (W m^{-2})	AWS2	LPNET14	–300 to +300 W m^{-2}	±5 %

Table S2: Specifications of the sensors used in automatic weather stations
